# Extra benefit of microalgae in raw piggery wastewater treatment: pathogen reduction

**DOI:** 10.1186/s40168-022-01339-3

**Published:** 2022-08-31

**Authors:** Sang-Ah Lee, Minsik Kim, Hee-Sik Kim, Chi-Yong Ahn

**Affiliations:** 1 Cell Factory Research Center, Korea Research Institute of Bioscience and Biotechnology (KRIBB), Daejeon, 34141 Republic of Korea; 2 Department of Environmental Biotechnology, KRIBB School of Biotechnology, University of Science and Technology (UST), Daejeon, 34113 Republic of Korea; 3 Environmental Safety Group, Korea Institute of Science and Technology (KIST) Europe, 66123 Saarbrücken, Germany

**Keywords:** Piggery wastewater, Microalgae, Metagenome analysis, Pathogens, Network analysis

## Abstract

**Background:**

Monitoring microbial communities especially focused on pathogens in newly developed wastewater treatment systems is recommended for public health. Thus, we investigated the microbial community shift in a pilot-scale microalgal treatment system for piggery wastewater.

**Results:**

Microalgae showed reasonable removal efficiencies for COD and ammonia, resulting in higher transparency of the final effluent. Metagenome and microbial diversity analyses showed that heterotrophic microalgal cultivation barely changed the bacterial community; however, the mixotrophic microalgal cultivation induced a sudden change. In addition, an evaluation of risk groups (RGs) of bacteria showed that raw piggery wastewater included abundant pathogens, and the microalgal treatment of the raw piggery wastewater decreased the RG2 pathogens by 63%. However, co-cultivation of microalgae and the most dominant RG2 pathogen, *Oligella*, showed no direct effects between them.

**Conclusions:**

Thus, a microbial interaction network was constructed to elucidate algae-bacteria interrelationships, and the decrease in *Oligella* was indirectly connected with microalgal growth via *Brevundimonas*, *Sphingopyxis*, and *Stenotrophomonas*. In a validation test, 3 among 4 connecting bacterial strains exhibited inhibition zones against *Oligella*. Therefore, we showed that microalgal wastewater treatment causes a decrease in RG2 bacteria, which is an indirect impact of microalgae associated with bacteria.

**Graphical Abstract:**

Video abstract

**Supplementary Information:**

The online version contains supplementary material available at 10.1186/s40168-022-01339-3.

## Introduction

Wastewater is a part of daily life, but recently, concerns about wastewater risks have been rising as bacteria in wastewater could affect human health [1]. Pathogens such as bacteria and viruses are abundant in raw wastewaters; hence, it is believed that pathogens must be continuously monitored to protect the public from possible outbreaks of diseases [2]. As harmful traits such as antibiotic-resistance genes could be shared between microbial communities [3], a subsequent removal process will not be effective once a group of harmful bacteria becomes dominant. Thus, pathogens in wastewater need to be examined using high-throughput technologies during treatment [4].

For most wastewaters that are generated near metropolitan areas, pathogen-related problems have been solved by adopting disinfection techniques in large-scale wastewater treatment plants. For instance, municipal wastewater is treated with UV or chemicals (peracetic acid, chlorine, or ozone) [5]. In contrast, livestock wastewater is generated in rural areas. Piggery wastewater is rich in nutrients, such as ammonium and organic matter; therefore, primitive treatment systems are often employed, such as composting, to utilize the nutrients for agriculture [6]. However, the composting of piggery wastewater generates a range of gaseous contaminants, such as ammonia, which can be released during storage, treatment, and disposal of waste [7], and emit greenhouse gases such as CO_2_ and methane [8].

Recent studies on wastewaters have focused on valorizing wastewater into reusable or valuable chemicals without greenhouse gas emissions using a biological conversion process [9]. Among the various emerging technologies, one of the most promising is microalgal wastewater treatment. Microalgae grow by photosynthesis, which utilizes CO_2_ and generates oxygen as a byproduct [10]. Furthermore, algae are suitable for nutrient removal and can swiftly adapt to stress conditions, such as excessive amounts of ammonium in raw piggery wastewater [11]. Consequently, many studies have utilized microalgae to treat raw piggery wastewater and achieved reasonable nutrient removal rates [12].

However, the previous studies have not investigated changes in pathogenic bacteria at the metagenomic level during microalgal wastewater treatment. Earlier studies regarding pathogens in microalgal wastewater treatment determined the pathogenicity of microorganisms by morphology and focused on coliform decay during the wastewater treatment process [13, 14]. These studies focused on physicochemical changes in wastewater, such as pH, temperature, and light, to elucidate the decrease in target bacteria, but the understanding of the interactions between bacteria and microalgae is still limited. On the other hand, most metagenomic studies on microalgae-bacteria interactions aimed at enhancing productivity; thus, they focused on growth-promoting bacteria or lipid-enhancing bacteria [15]. In a microbiome study on microalgal piggery wastewater treatment, no pathogens were detected because the feedstock was obtained from stabilization lagoons [16]. Although relationships between microalgae and bacteria in wastewater are poorly understood because of the complexity of the interactions between biota [17], recent developments in statistical correlation analyses have enabled the elucidation of microbial communities at higher resolution and their potential interactions [18]. As pathogens and microbial changes are an emerging issue [19] and biological treatment causes a microbial community change, metagenome analysis is required to investigate changes in pathogenic bacteria during the microalgal piggery wastewater treatment process. Furthermore, to validate the possibility of industrialization of any newly developed processes, an experiment should be conducted preliminarily on a pilot scale. However, most previous studies on community analysis have been conducted using small-scale laboratory bioreactors [20].

The present study focused on bacterial community changes during the microalgal treatment of piggery wastewater at a pilot scale. Undiluted raw piggery wastewater was treated using a two-step treatment process developed in our previous study [21]. Amplicon sequencing was used to reveal the shift patterns of the bacterial communities. To further examine the microalgal effects on pathogens, bacteria were classified by risk group (RG), a set of biocontainment precautions. The bacterial community changes were analyzed using various statistical methods, and the direct and indirect interactions between bacteria, environmental factors, and microalgal growth were analyzed by co-cultivation testing and network analysis, respectively.

## Methods

### Preparation for seed culture and pilot-scale photobioreactor

*Coelastrella* sp., a microalga isolated from ammonia-rich wet soil near a swine farm [11], was utilized for piggery wastewater treatment. To complete the two-step piggery wastewater treatments, 1.0 g L^−1^ of initial biomass concentration was required to endure ammonia stress of piggery wastewater; therefore, seed cultivation was gradually scaled up. Furthermore, pilot-scale algal seed cultures were maintained for a sustainable piggery wastewater treatment process. The first seed was incubated in a 250-mL flask filled with 100 mL of BG-11 medium [22] in a thermostatic room at 25 °C. Subsequently, two 10-L photobioreactors (PBRs) were prepared as intermediate seed cultures in the same room. Before the intermediate seed cultivation, the reactors were filled with BG-11 medium and autoclaved at 121 °C for 60 min. *Coelastrella* sp. was inoculated at 0.2 g L^−1^ for a seed cultivation. The light was irradiated by LED jackets, and the light intensity was gradually increased from 800 μmol m^−2^ s^−1^ to 1800 μmol m^−2^ s^−1^ along with an increase in microalgal biomass. The cultures were aerated with 5% CO_2_ at 0.6 vvm and stirred at 105 rpm with magnetic stirrer bars. After reaching 3.0 g L^−1^ of dry cell weight (DCW), the culture broths were transferred to a 250-L pilot-scale PBR filled with 200 L of BG-11 medium. Before transferring the seed culture, all pilot-scale PBRs were sterilized by 230-fold diluted NaClO for 24 h. Sparge rings were installed to generate microbubbles at the bottom of the PBR, and the airflow rate was kept at 0.3 vvm with 5% CO_2_. The pilot seed culture utilized sunlight at outdoor air temperature.

### Pilot-scale piggery wastewater treatment using microalgae

**Fig. 1:**
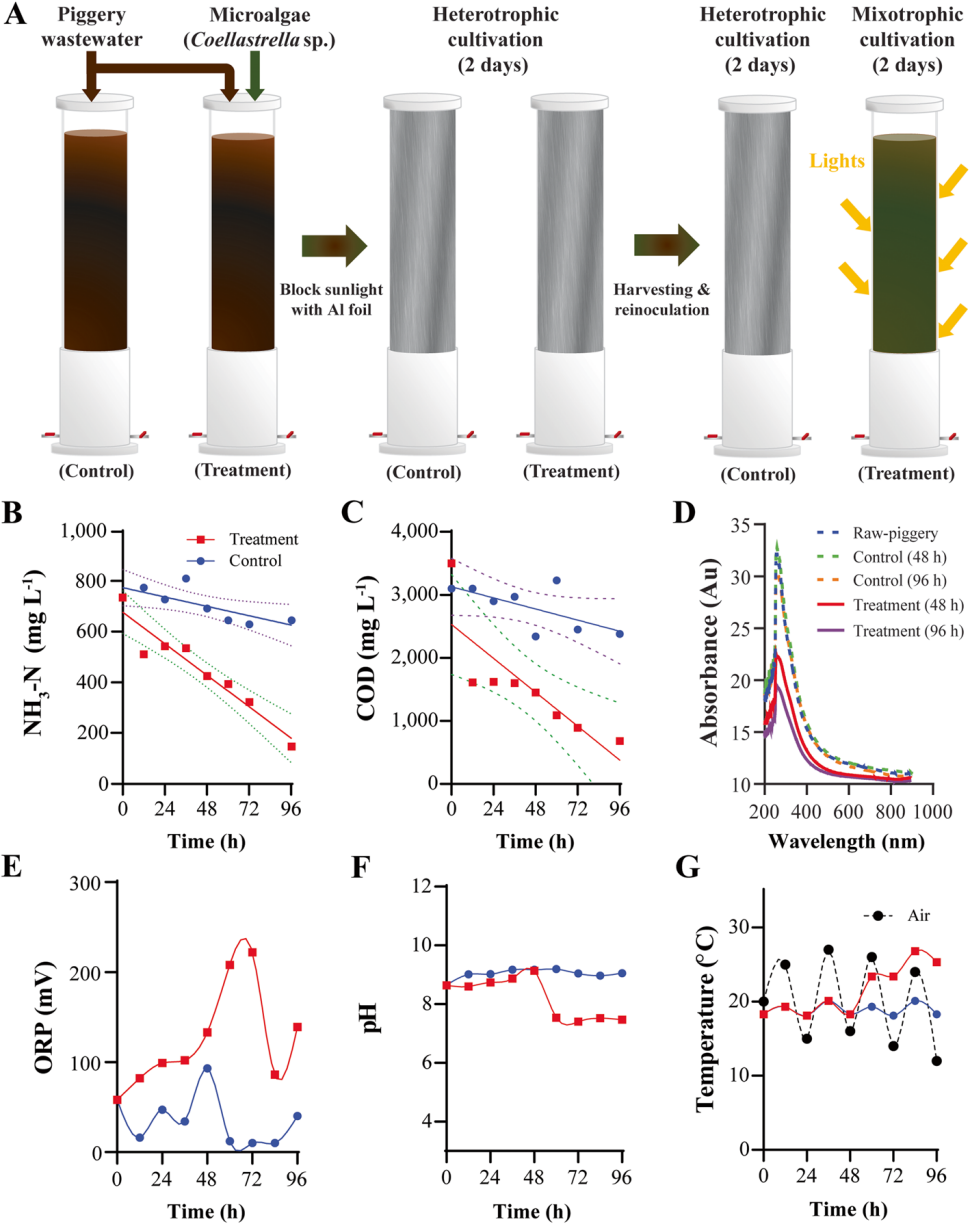
Methodology and results of piggery wastewater treatment using microalgae. **A** Experimental scheme of wastewater treatment, **B** changes of ammonia concentration, **C** chemical oxygen demand (COD) with 95% confidence intervals, **D** absorbance, **E** oxygen-reduction potential (ORP), **F** pH, and **G** temperature.

To treat raw piggery wastewater efficiently, a two-step (heterotrophic and mixotrophic) microalgal cultivation strategy was employed based on our previous study [21]. For the heterotrophic phase of wastewater treatment, pilot-scale PBRs were covered with aluminum foil to block natural sunlight, and then fresh raw piggery wastewater from Jacob Farms Corporation in South Chungcheong Province were transferred to the PBRs. The initial composition of the raw piggery wastewater is shown in Table S1. Thickened microalgal seed culture was prepared by pausing agitation for 6 h and transferred to PBR through a sampling port using a centrifugal pump. After 48 h of heterotrophic cultivation, the biomass was collected using the same sedimentation method, and fresh microalga seeds were re-inoculated into the reactor for the mixotrophic phase of wastewater treatment. In this step, the aluminum foil was removed for algal photosynthesis. The initial algal biomass was 1.0 g L^−1^ in both cultivation steps. In the outdoor mixotrophic process, natural sunlight irradiated the PBR during the daytime, and LED panels (GODOX, China) supplemented with 2600 μmol m^−2^ s^−1^ of irradiation were applied to the PBR for 48 h. As a control, an aerobic digestion process was conducted under the same air flow rate for 96 h. The control group was grown in the dark without inoculation with any microorganism, as shown in Fig. 1A. During the cultivation process, 100 mL of the samples was collected every 12 h for further analyses.

### Metagenomic analysis of bacterial communities via next-generation sequencing

To analyze the bacterial community changes during the microalgal treatment of piggery wastewater, 2 mL of each sample was filtered through a 0.22-μm sterile polycarbonate membrane filter (GSWP04700, Millipore Corp., USA). All filtered samples were preserved in a – 80 °C deep freezer before extracting the microbial genomic DNAs (gDNAs). After collection of the final sample, bacterial gDNAs of all stored filters were extracted using a ChargeSwitch Forensic DNA Purification Kit (CS11200, Thermo Fisher Scientific Inc., USA). Partial bacterial 16S rRNA genes in the extracted gDNAs were amplified using 341F (5′-CCTACGGGNGGCWGCAG-3′) and 805R (5′-GACTACHVGGGTATCTAATCC-3′) primers targeting the V3-V4 regions for amplicon sequencing [23]. PCR amplification was conducted using Ex Taq^TM^ Hot Start Version (RR006A, Takara Bio Inc., Japan), and PCR products were purified using AMPure XP beads (A63881, Beckman Coulter, USA). Sequencing was performed using a MiSeq system (Illumina Inc., USA), which is a high-throughput paired-end sequencer, by outsourcing to Macrogen (Republic of Korea).

Metagenome data from MiSeq was used to investigate amplicon sequence variants (ASVs). Barcoded 16S rRNA genes were defined by DADA2 [24] according to the DADA2 pipeline tutorial v1.8 (https://benjjneb.github.io/dada2/tutorial_1_8.html). Each bacterium was assigned to genus, family, order, class, phylum, and kingdom levels with Silva v138.1, which is a DADA2-formatted reference database (https://benjjneb.github.io/dada2/training.html). Species were assigned only when an ASV matched 100% with the Silva species assignment database v138.1 [25]. After the classification of sequences, chloroplast and mitochondrial data were removed to avoid misinterpretation [26]. The raw sequences and metadata were submitted to the National Center for Biotechnology Information (NCBI) Sequence Read Archive (SRA). The project number is PRJNA757236.

### Analyses of major nutrients and microelements

The ammonia nitrogen (NH_3_-N), total phosphorus (TP), and chemical oxygen demand (COD) were analyzed using water quality analysis kits (10313-NH_3_-N/10623-TP/10111-COD, C-mac Co., Ltd., Korea). Concentrations of B, Na, Mg, K, Ca, Fe, Co, Cu, and Mo were analyzed by inductively coupled plasma-mass spectrometry (ICP-MS) using an Agilent 7700s (Agilent, USA) at the Korea Basic Science Institute (KBSI).

### Relative absorbance of the effluent

The relative absorbance was measured to digitize the decolorization effect of microalgal treatment on piggery wastewater. Each sample was 10-fold diluted and transferred to UV-quartz cuvettes (C2003P1, Ossila, UK). Absorbance was measured using a UV-visible spectrophotometer (S-3100, Shimadzu, Japan).

### DCW measurement

Microalgal growth in every cultivation system was directly measured in DCW by the gravimetric method [11]. For statistical analyses, algal growth (accumulated values of the DCW increase) was utilized, as biomass was harvested twice at the end of both heterotrophic and mixotrophic cultivation.

### Diversity analysis

The changes in the microbial communities of the control and algal treatment groups were appraised throughout the wastewater treatment by various diversity indices. Shannon-Weaver, Simpson’s, and inverse Simpson indices were calculated using vegan 2.5-7 (https://cran.r-project.org/web/packages/vegan/vignettes/diversity-vegan.pdf). Menhinick’s diversity index was calculated based on the equation in an earlier study [27].

### Coordinate multi-dimensional analyses

Principal coordinates analysis (PCoA) and distance-based redundancy analysis (db-RDA) were performed to summarize the ordination of the environmental factors and microbial community changes. RDA, capscale, and vegdist functions in vegan 2.5-7 were utilized for the calculations.

### Assignment of pathogenicity

To investigate the pathogenicity of bacteria, the RGs of organisms were identified according to Singh et al. [28] at both the genus and species levels using the RG database at the American Biological Safety Association (ABSA) (https://my.absa.org/Riskgroups). Every organism was classified as RG1 (not associated with disease in healthy adult humans) or RG2 (associated with human disease, which is rarely serious and for which preventive or therapeutic interventions are often available). The detailed pathogenicity of species-assigned taxa was identified by searching for species information. Genus-level pathogenicity was determined by searching the database for genera that contain “spp.” (several unspecified species) as RG2 pathogens.

### Isolation of *Oligella spp.* from the raw piggery wastewater

To isolate *Oligella* spp., the raw piggery wastewater was serially diluted with sterile saline solution (10^−5^ to 10^−6^), and 100 μL aliquots of each dilution were spread on Luria-Bertani (LB), trypticase soy agar (TSA), and Reasoner’s 2A agar (R2A) media plates [29]. Grown colonies were picked and cultivated with trypticase soy broth (TSB) medium in a cultivation chamber at 25 °C for four days. Successful cultures were preserved as stocks with the same medium containing 20% glycerol (v/v) in a –80 °C freezer. The strain information was identified by amplifying and sequencing their whole 16S rRNA region and constructing a phylogenetic tree by the neighbor-joining method and Kimura 2-parameter model using MEGA X [30].

### Real-time PCR

To examine the growth inhibition effect on bacteria of microalgae, a co-cultivation test was conducted with filter-sterilized piggery wastewater in a 0.22-μm bottle-top filter (430758, Corning, USA), and changes in *Oligella* were observed by qPCR analysis. *Coelastrella* sp. and *Oligella* were inoculated into filtered piggery wastewater, whereas *Coelastrella* sp. was not added to the control group. The gDNAs of daily samples were extracted using the FastDNA SPIN Kit for Soil (116560200, MP Biomedicals, USA). For the absolute quantitation of *Oligella*, a specific primer set was designed from the whole 16S rRNA sequence of the *Oligella* strain isolated in this study (F: 5′-CCAGCAGCCGCGGTAATACA-3′; R: 5′-TACCCACGCTTTCGTGCCTG-3′). The absolute quantitation of *Oligella* was calculated based on the standard calibration points, and its relative abundance was determined using the absolute quantity of genes identified by 341F/805R primers.

### Disk diffusion test

The disk diffusion test was performed using the Kirby-Bauer protocol [31]. First, bacterial cells were cultured in a baffled flask at 30 °C for three days in TSB medium. *Oligella* culture diluted to 0.2 OD_600_ was pre-spread in TSA plates with sterile cotton swabs in advance. Then, 10-mm filter paper disks were impregnated with 100 μL of culture broth or supernatant and were deposited on the plates. The filter papers of the negative control contained the same volume of distilled water. Growth on the plates was monitored at 25 °C under light panels for 2–4 days. The following four bacterial disk strains were acquired from the Korean Collection for Type Cultures (KCTC) and cultured in the sample medium: *Brevundimonas terrae* (KCTC 12481), *Sphingopyxis panaciterrae* (KCTC 22112), *Stenotrophomonas koreensis* (KCTC 12211), and *Stenotrophomonas ginsengisoli* (KCTC 12539). The concentrations of bacteria for disks were adjusted to 0.2 OD_600_ with 0.85% saline solution before use in the test. After identifying the inhibition zones of *Oligella*, the diameters were measured using a Vernier caliper.

### Network analysis

To investigate the direct and indirect relationships between ASVs and environmental factors, Spearman’s rank correlation coefficients were calculated using the Hmisc package in R. The data were trimmed by values with *p-BH* < 0.001 (Benjamini-Hochberg *p*-values) and |*ρ*| > 0.88 (correlation coefficient). For a further filtration process to prune relatively irrelevant correlations, a core network was constructed only with species with a maximum relative abundance level higher than 0.2%. The calculated correlations were visualized using the open-source program Cytoscape version 3.8.2 [32].

## Results

### Pilot-scale piggery wastewater treatment using microalgae

To verify the effect of microalgal treatment on undiluted piggery wastewater at a pilot scale of 250 L, outdoor cultivation systems were prepared. Sequential heterotrophic (48 h) and mixotrophic (48 h) microalgal cultivation using piggery wastewater was conducted. As a control group, aerobic digestion was conducted without adding microalgae to the PBR for 96 h. The experimental scheme and the main results are shown in Fig. 1. Compared to the aerobic digestion group (control), the microalgal treatment reduced ammonia more significantly during the 96 h of reaction (Fig. 1B). While there was only a 12.3% decrease in ammonia in the control group, over 80.1% of ammonia was eliminated with microalgae, resulting in a 6.5-fold increase in removal efficiency. Similarly, the total COD decrease in the treatment and control groups differed by 80.6% and 23.3%, respectively (Fig. 1C). Heterotrophic cultivation during the first 48 h of treatment decreased the COD by 54.0% within 6 h, and further heterotrophic cultivation was not able to reduce residual COD. The other physical or chemical properties of both experimental groups were different. The absorbance of wastewater decreased significantly during the treatment process, especially at a wavelength of approximately 260 nm, while the absorbance of the control group did not show a significant decrease (Fig. 1D). The maximum relative absorbance of raw piggery wastewater was 32.14 at 256 nm. The maximum absorbance in the control group slightly decreased to 30.19, but the treatment group showed a greater decrease, resulting in a decrease to 21.14. The increased transparency of the microalgae-treated wastewater was confirmed by the naked eye. The oxidation-reduction potential (ORP) increased in the treatment group (Fig. 1E), while the control group showed consistently low ORP. The increasing trend of ORP is usually known to indicate the production of oxygen. The pH drastically decreased at the beginning of the mixotrophic cultivation process (Fig. 1F). Because of the nature of the outdoor experiment, the temperature during wastewater treatment fluctuated with the daily temperature changes in both systems until day 2 (Fig. 1G). However, the temperature of the treatment group in the mixotrophic mode (48–96 h) increased significantly because of the solar energy and additional light sources. Furthermore, the concentrations of the initial and final residual micronutrients, that is, B, Na, Mg, K, Ca, Fe, Co, Cu, and Mo, were analyzed (Table S1). B, Mg, K, and Fe decreased with the microalgal treatment. In contrast, the concentrations of Na and Cu increased in both experimental groups. The Ca concentration in the treatment group increased, but that of the control group decreased.

### Changes in bacterial communities during treatment of piggery wastewater

**Fig. 2:**
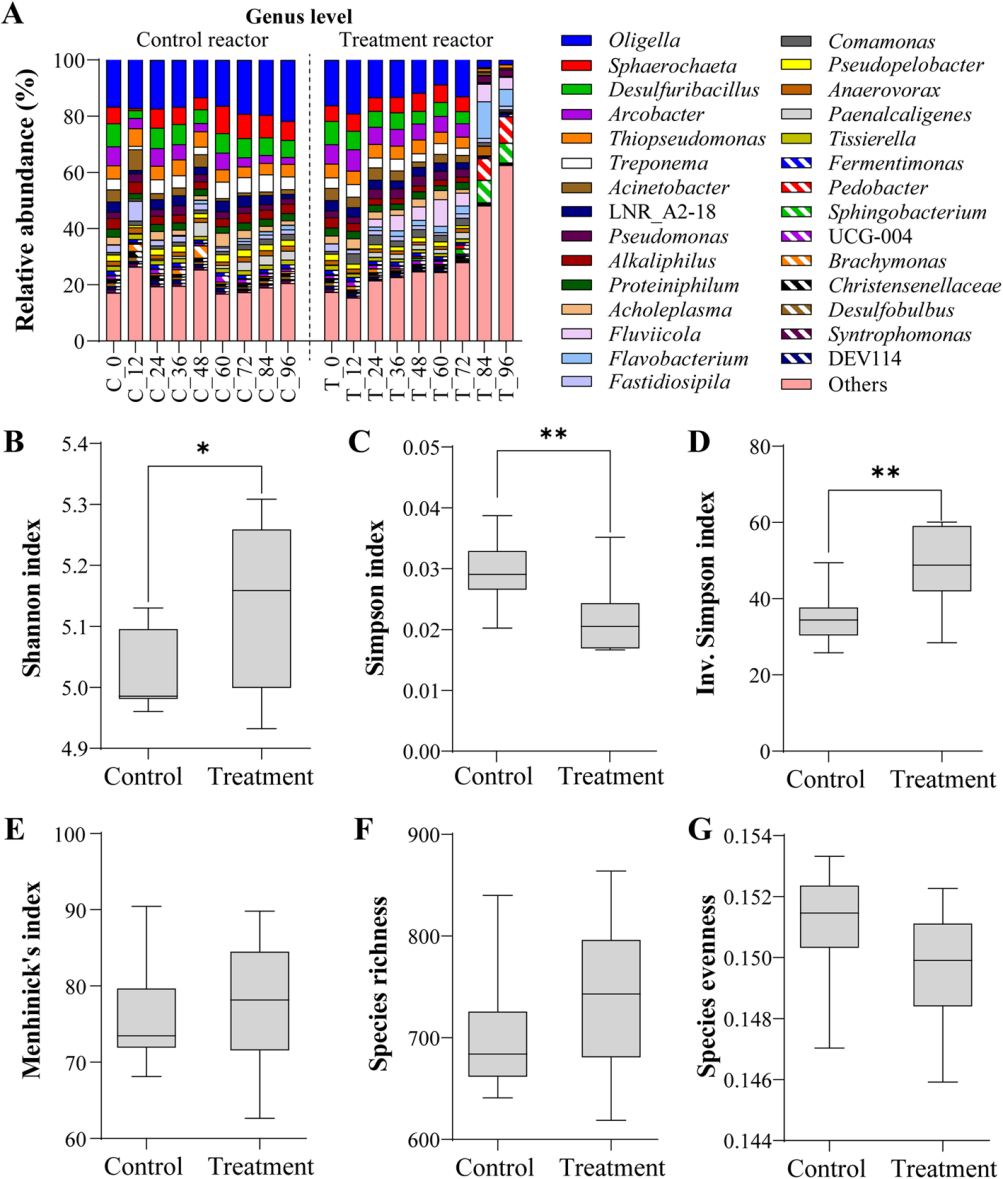
Results of next-generation sequencing in amplicon sequence variant (ASV) reads of control and microalgae-treated group. **A** Relative abundances of the 30 most abundant genera in control and treatment; and box charts of various diversity indices for **B** Shannon index, **C** Simpson index, **D** Inverse Simpson index, **E** Menhinick’s index, **F** species richness, and **G** species evenness at significance level of *p < 0.05 and **p < 0.01

During the wastewater treatment process, changes in the bacterial communities were analyzed based on amplicon sequencing. The relative abundances in the family, order, and class levels are shown in Figure S1–3. A total of 3035 ASVs were assigned at the genus level, and among them, 87 ASVs were assigned at the species level. The composition of microbial communities in the control group showed no significant changes during the entire reaction time (Fig. 2A). In contrast, the microalgal treatment group showed slight changes in microbial composition until 72 h of reaction, and the community had drastically changed at 84 h. This trend continued until the end of the experiment. At the end of the microalgal treatment, the proportion of initially abundant genera commonly found in the control decreased, but other genera replaced them. From the results, *Coelastrella* sp., a high ammonia-adapted strain, was confirmed to affect the bacterial community structure. Alpha diversities were calculated using the Shannon index (Fig. 2B) and Simpson’s index (Fig. 2C, D). Both indices verified the differences in microbial communities between the control and treatment groups. The microbial community in the microalgal treatment group changed more drastically over time compared to the control, towards increasing its alpha diversity. Menhinick’s index (Fig. 2E) and species richness (Fig. 2F) showed that the alpha-diversities increased in the treatment group compared to the control, but the differences were not statistically significant. Species evenness (Fig. 2G) decreased in the treatment but it was not statistically significant.

### Statistical analyses of microbial community and environmental factors

**Fig. 3:**
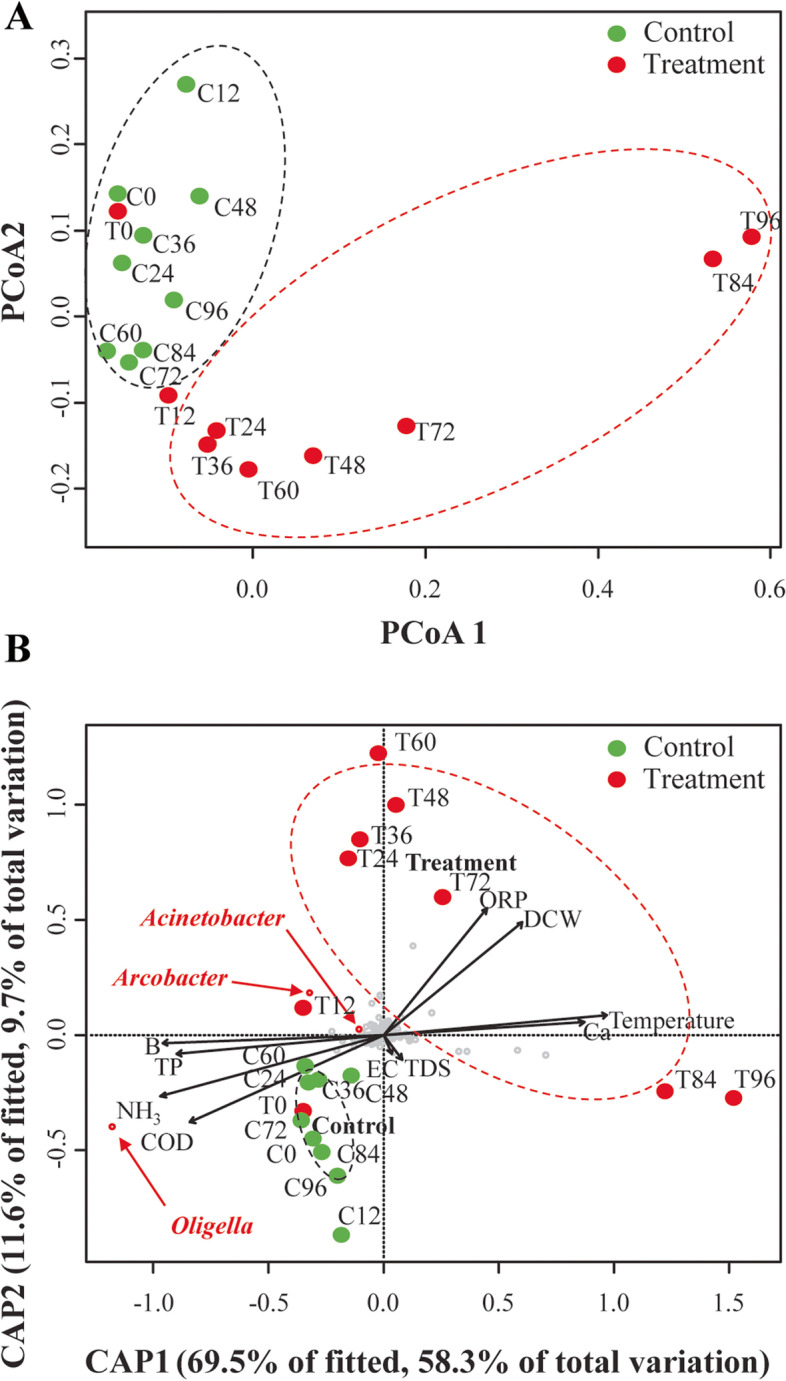
Ordination diagrams of environmental factors using **A** principal coordinate analysis (PCoA) and **B** distance-based redundancy analysis (db-RDA) based on canonical analysis of principal coordinate (CAP) plots using Bray–Curtis distance. Each graph shows the difference between the microalgae-wastewater treatment group and the control group

A PCoA result with clustering is shown in Fig. 3A to compare and visualize the differences in bacterial communities. The PCoA coordinates of both groups were placed adjacent to each other at the beginning of the treatment process. While every coordinate of the control group was concentrated near the point of 0 h, the PCoA plot of the treatment group moved gradually out of the control group-concentrated area along the processing time. Consequently, the point of treatment at 96 h was located at the most distant point from 0 h. To investigate the correlation between the microbial changes in ASVs and environmental factors, such as DCW of microalgae, temperature, pH, ORP, COD, and micronutrients, db-RDA was conducted (Fig. 3B).

### Changes of pathogens (RG2) during the wastewater treatment

**Fig. 4:**
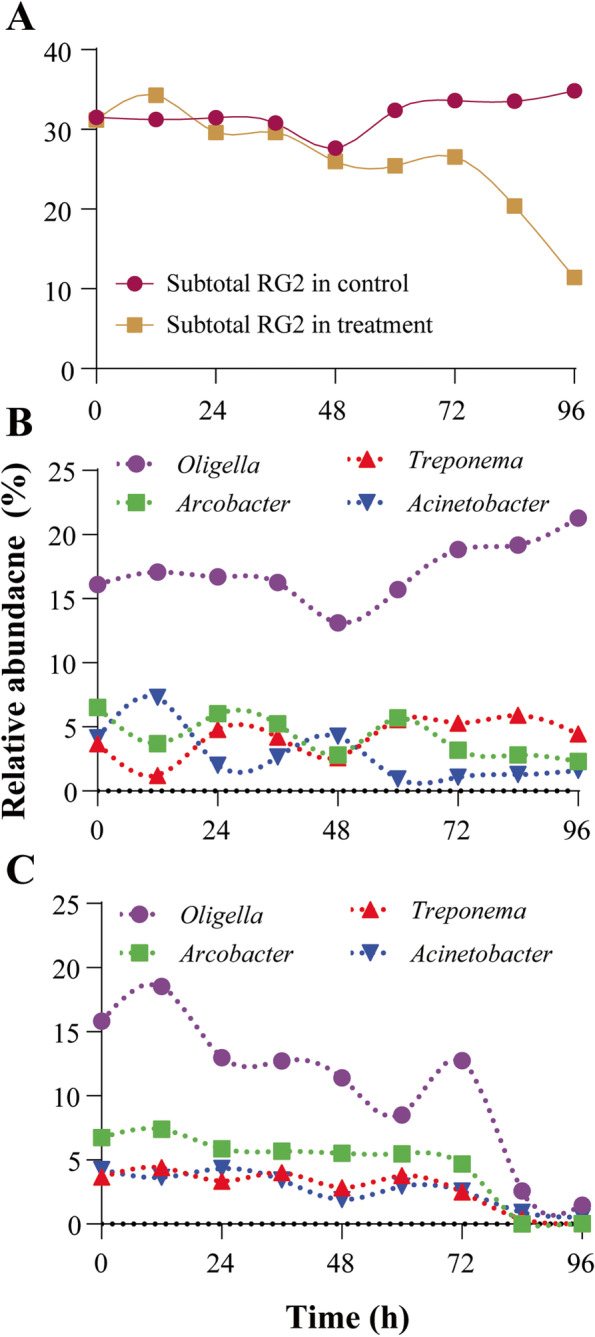
**A** Subtotal relative abundances of Risk Group 2 (RG2) (429 ASVs, 28 genera) in the control and microalgal treatment. **B** Relative abundances of the four most abundant genera in RGs of the control group and **C** microalgal treatment group

The population change of pathogens over time in the microalgal piggery wastewater treatment system was analyzed (Fig. 4A). Because the proportions of RG2 were determined at the genus level, the numbers of RG2 may have been exaggerated, as every species that matched the RG database with spp. (several species) was considered pathogens. The differences in relative abundance of RG2 between the two experimental groups were significant, particularly during the later period of the experiment. The subtotal relative abundance of RG2 in the control group did not show significant changes, whereas that in the treatment group decreased from 31.2 to 11.4%. Further analysis of the changes in these four pathogenic genera was conducted. The relative abundance of pathogenic bacteria in the control group showed inconsistent minor changes during the entire cultivation period (Fig. 4B). The summation of the proportion of the four pathogenic bacteria in the control group at 0 h and 96 h resulted in 30.5% and 29.7%, respectively, remaining constant. On the other hand, these four pathogenic bacteria drastically decreased in the microalgae-treated group (Fig. 4C). *Oligella* decreased from 15.8 to 1.5%, *Arcobacter* decreased from 6.76 to 0.03%, *Treponema* from 3.66% to 0.07%, and *Acinetobacter* from 4.26 to 0.56%. Therefore, the proportion of four pathogenic bacteria over the total ASV decreased drastically from 30.5 to 2.1% after 96 h of microalgal treatment. Among RG2, the genus *Flavobacterium* showed a significant increase in the treatment reactor, from 0.04 to 5.99%, indicating a possible increase of pathogens in the microalgal treatment (Fig. 2A). However, most previous studies on the pathogenicity of *Flavobacterium* focused on *F. psychrophilum*, a fish pathogen [33]; hence, they were not considered in this study.

### Analyses of interactions between microalgae and bacteria

**Fig. 5:**
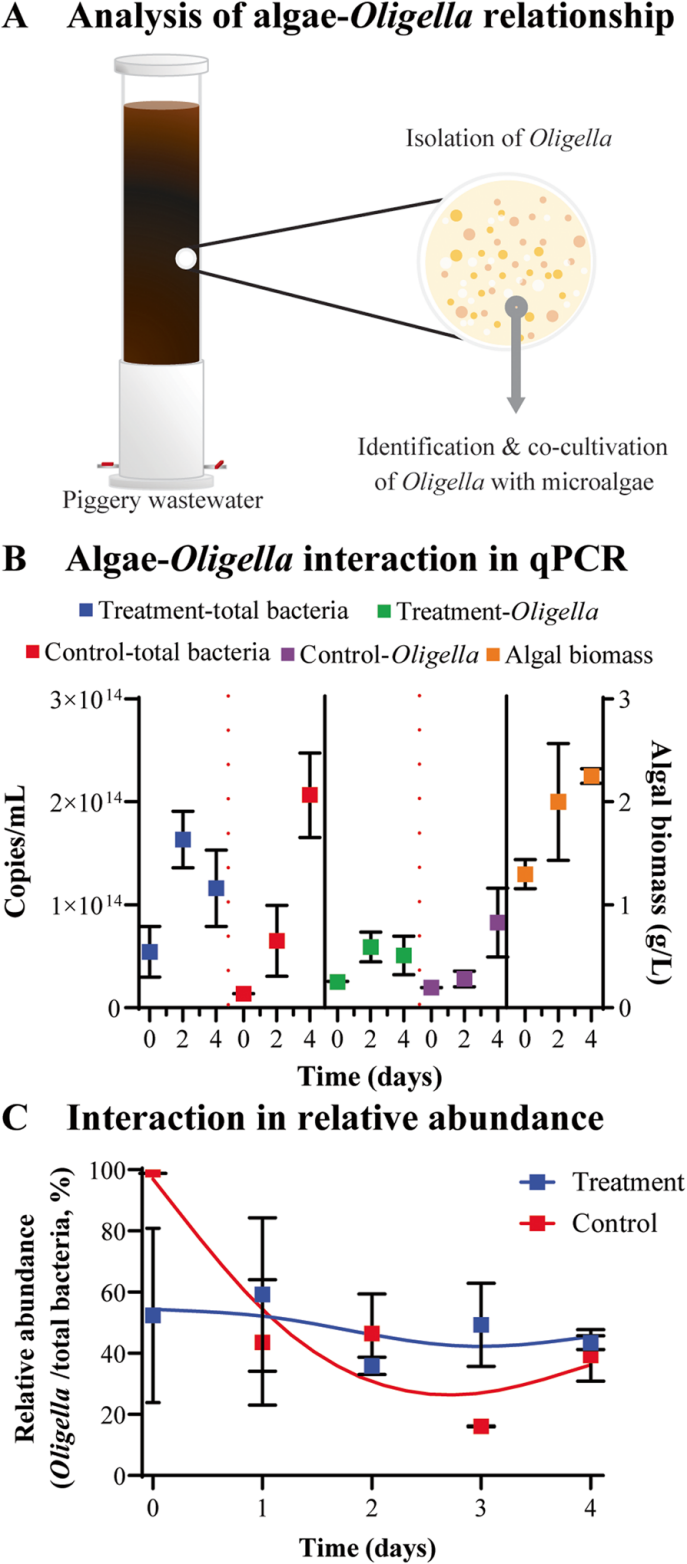
**A** Experimental schemes to examine the relationship between *Coelastrella* sp. and *Oligella* sp. **B** *Coelastrella*-*Oligella* co-cultivation results in copy numbers of total bacteria, that of *Oligella*, and biomass of *Coelastrella*, identified by global 16S rRNA qPCR, *Oligella*-specific 16S qPCR, and dry cell weight, respectively. **C** Relative abundance changes of *Oligella* by time in the co-cultivation test

**Fig. 6:**
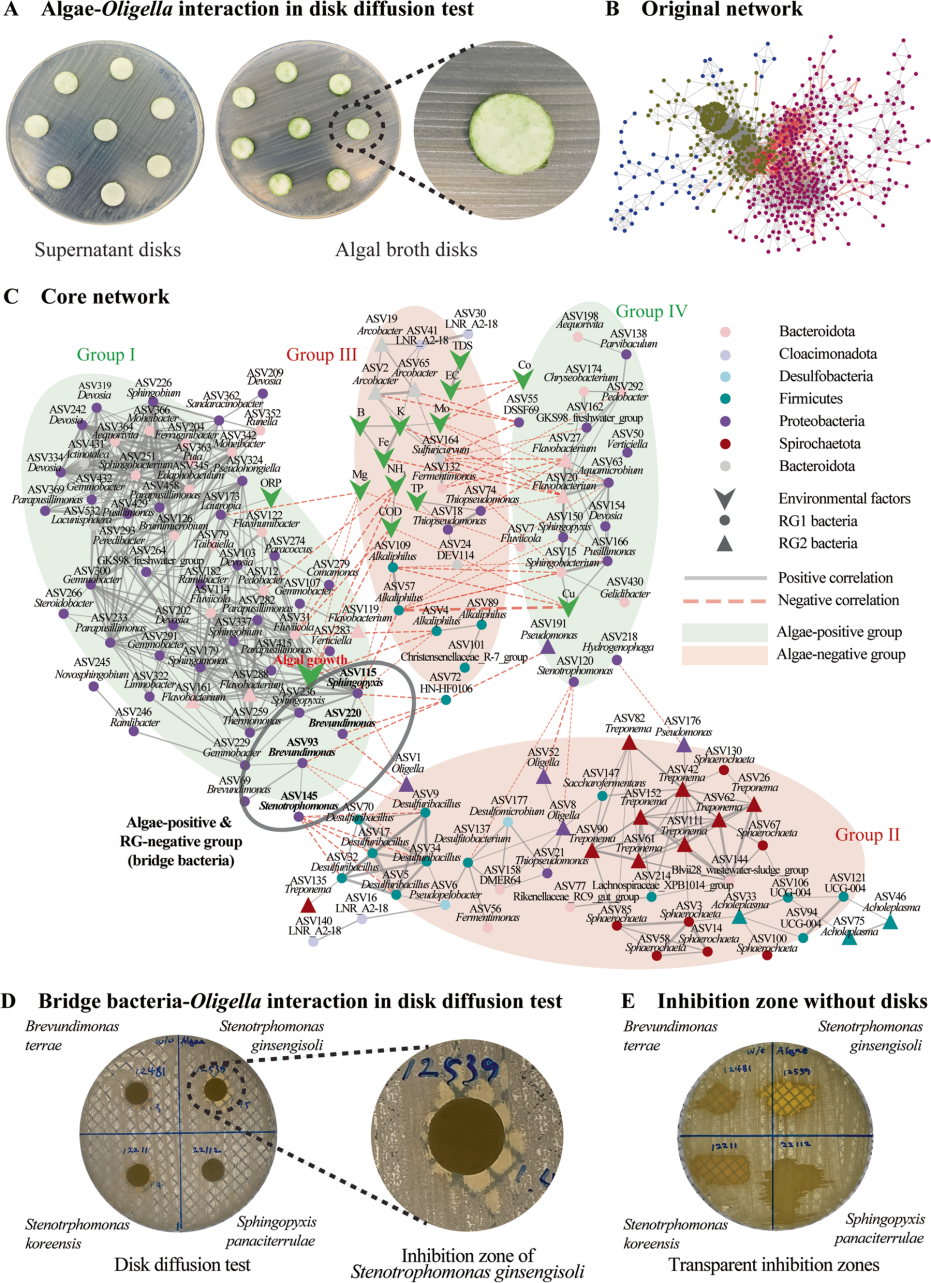
**A** Results of disk diffusion test for direct interaction between *Coelastrella* sp. and *Oligella* sp. **B** A network of amplicon sequencing variants (ASVs) and environmental factors in pilot-scale wastewater treatment and **C** a core network of abundant species (over 0.2%) filtered with statistical significance at *p_BH* < 0.001 and |*ρ*| > 0.88. **D** Results of disk diffusion tests for interaction between *Oligella* sp. and four bridge bacteria identified by network analysis. **E** Inhibition zones without disc

To validate the relationship between algal growth and pathogen decrease, several *Oligella* spp., the most abundant genus in the raw piggery wastewater, were isolated, and a strain closest to ASV1 in a phylogenetic tree (Figure S4) was co-cultivated with *Coelastrella* sp. (Fig. 5A). Changes in the absolute amount of *Oligella* and other bacteria were measured by qPCR (Fig. 5B). The qPCR efficiency was 93.98%. It seems that the total of quantified genes of *Oligella* grown with microalgae was smaller than that of the control group. However, as the total number of bacteria increased, the relative abundance of *Oligella* among total bacteria in the treatment group showed no difference relative to the control on day 4 (Fig. 5C). A disk diffusion test was conducted to visualize the inhibitory effect of microalgae on *Oligella*, but it showed that *Coelastrella* sp. did not directly inhibit the growth of *Oligella* sp. (Fig. 6A). Therefore, an interaction network of microbes was constructed based on Spearman’s rank-order correlation to identify microalgae-bacteria relationships (Fig. 6B). A core network was also constructed by cutting species abundance (over 0.02%) (Fig. 6C). ASVs and environmental factors were grouped into four clusters according to their correlation with algal growth. The algal growth and growth of pathogens were not directly correlated in the network at *p_BH* < 0.001, but were identifiable at a higher threshold, for example, between ASV1 and algal growth at *p_BH* < 0.005. However, they were not included in the network analysis because of the more significant correlations. The majority of RG2 was located in the algae-negative groups (groups II and III). Group I had strong negative correlations with group II, such as *Sphingopyxis*-*Oligella*, *Brevundimonas-Oligella*, *Brevundimonas-Desulfuribacillus*, and *Stenotrophomonas*-*Desulfuribacillus*. Group III, which included three RG2 ASVs (*Arcobacter*), seemed to decrease along with environmental factors such as TP, K, and Fe. Group IV was related to Group I with negative-negative correlations; therefore, both were considered as algae-positive groups and contained six RG2 ASVs, namely five *Flavobacterium* and one *Pseudomonas*. However, *Flavobacterium* usually harms fish but not humans [34], and the increase in *Pseudomonas* was minimal (0.06 to 0.21%).

As Group II-bordering bacteria in group I may have acted as key players in suppressing Group II, a literature survey was conducted on *Brevundimonas* (ASV93 and ASV220), *Sphingopyxis* (ASV115), and *Stenotrophomonas* (ASV145). *Brevundimonas* exhibited antimicrobial activity against microalgae when associated with AgNO_3_ in an earlier study [35]. The switching of relative abundances between *Stenotrophomonas* and *Oligella* was identified from the data of a previous study [36]. *Sphingopyxis* is capable of degrading xenobiotics and other environmental contaminants and is able to produce ectoine [37], an osmoprotectant that can negatively affect other microorganisms by changing osmolality.

Based on the above information, these bacteria were used for the disk diffusion test for *Oligella*. Three bacterial species exhibited clear zones by inhibiting *Oligella* growth, while the control group (microalgae) and *Sphingopyxis* did not (Fig. 6D). The inhibitory zone diameters of *Brevundimonas terrae*, *Stenotrophomonas ginsengisoli*, and *Stenotrophomonas koreensis* were similar (Table S3). A further test with bacterial broth dropped on *Oligella* colonies showed that these three bacterial species efficiently suppressed *Oligella* (Fig. 6E).

## Discussion

The COD of piggery wastewater can be divided into two fractions: (1) biodegradable COD and (2) non-biodegradable COD, with a ratio of 8:2 (data not shown). Therefore, most biodegradable COD was assimilated after sequential treatment processes using microalgae. However, the COD reduction pattern of the treatment reactor showed a stepwise decrease. Organic matter can be consumed promptly by microalgae immediately after inoculation, because heterotrophic carbon uptake is generally faster than CO_2_ uptake by photosynthesis. Such a rapid decrease in COD could also be attributed to aggregation or adsorption to microalgae [38]. Mixotrophic cultivation at 48–96 h eliminated most of the residual biodegradable COD in 2 days. The increasing trend of ORP is usually known to indicate the production of oxygen [39]; therefore, microalgal photosynthesis was successful in the treatment reactor. The lowered ORP after 72 h of treatment could be attributed to the growth of algae-positive groups accompanying respiratory activities. The maximum temperature of the treatment reactor was 26.8 °C at 84 h, while that of the control group was 20.1 °C in the meantime. The temperature of both groups remained the same during heterotrophic cultivation, but it varied at the mixotrophic step because of sunlight. Although the maximum temperature difference between both groups was 6.7 °C, the average daily outdoor temperature difference of the atmosphere at the site during the experiment was 10.8 °C, which was higher than the maximum temperature difference of the treatment reactor. Various previous studies have investigated the microalgal behavior in piggery wastewater. Zhu et al. [40] optimized the growth of *Chlorella zofingiensis* in diluted piggery wastewater (initial COD, 1900 mg L^−1^). However, this study utilized undiluted piggery wastewater according to our previous studies [11, 21], and the initial concentrations of COD (3100 mg L^−1^) and ammonia-nitrogen (770 mg L^−1^) were much higher than the usual tolerance range for microalgae. Fernández-Linare et al. [41] achieved a higher removal rate at a pilot scale relative to this study, but it required 13 days of cultivation. Compared with earlier studies, the present study eliminated a similar amount of COD and ammonia in a pilot-scale PBR, resulting in a significant decrease in absorbance (i.e., increased transparency) over a wide range of wavelengths over 4 days. Considering that the initial microalgal inoculum was limited due to the size of the pilot-scale seed culture, the current study successfully removed pollutants from the undiluted wastewater within 4 days.

The decrease in Mg can be assumed to be a consequence of chlorophyll synthesis in the treatment group, since chlorophyll requires Mg as a central atom. Considering the mass balance, the increase in Ca in the treatment group could be induced by cell lysis of microalgae. Plus, CO_2_, which was continuously supplied to the reactor, could have reacted with Ca and formed CaCO_3_. The CO_2_ supplied to the treatment group was continuously consumed by microalgae via photosynthesis, whereas that in the control group accumulated. The increase of Na in both groups may have occurred by bacteria degrading organic materials; hence, further analysis of the decreased microbiomes is required. The metagenomic analysis also indicated that most ammonia-nitrogen removals were caused by microalgae, because nitrifying bacteria, that is, ammonia-oxidizing bacteria and nitrite-oxidizing bacteria [42], were not listed as major taxa. Among the nitrifying bacteria, the most abundant genus was *Nitrospira*, identified as ASV189, with a lower abundance (< 0.5%). The relative abundance of ammonia-oxidizing bacteria, such as *Nitrosomonas*, was minimal. As none of the nitrifying bacteria were dominant in the piggery wastewater, ammonia reduction was insufficient in the control group. However, 80% or more of the ammonia was reduced in the microalgal treatment group, mainly by the inoculated microalgae.

The diversity analysis results could show that the microalgal treatment process significantly increased the alpha diversity of the microbial community. The same tendencies were observed for PCoA and db-RDA, namely concentrated control groups and distributed treatment groups. Environmental factors were also grouped into bidirectional tendency: one group consisted of ORP, DCW, temperature, and Ca; the other group included COD, NH_3_, TP, and boron. As microalgae consume COD, NH_3_, and TP as major nutrient sources, the treatment group should have a negative correlation with these factors. Therefore, the drastic changes in the ASV of the bacterial community in the microalgal treatment group were verified based on the PCoA and db-RDA. EC and TDS were not significantly correlated with any other environmental factors or communities. Microalgal growth and increased temperature seem to have caused changes in microbial communities, wherein the temperature increase was inevitable in mixotrophic cultivation mode. In another study, microalgal treatment of piggery wastewater changed the microbial community; however, it utilized diluted piggery wastewater from lagoons, and the dilution effect may have affected the microbial communities differently from this study with undiluted wastewater [16].

Four genera among the seven most abundant genera belonged to RG2. The most abundant genus was *Oligella*, which includes *O. ureolytica* [43] and *O. urethralis* [44] and can infect the bloodstream or induce urosepsis, respectively. The fourth most abundant *Arcobacter* is considered to pose a risk to human health, causing enteritis [45]. The genus *Treponema* also has pathogenic species such as *T. pallidum*, whose subspecies are responsible for diseases such as syphilis, bejel, and yaws, and *T. carateum*, which is the cause of a skin disease pinta [46]. *Acinetobacter* spp. are considered nosocomial pathogens [47]. These bacteria were opposite to algal growth in the dbRDA (Fig. 3). Piggery wastewater can contain various pathogenic microorganisms derived from pig manure, and their species composition varies greatly according to handling practices, storage management, etc. [48]. Hence, developing cost-effective treatment methods for pathogen removal in piggery wastewater is urgently needed. Microalgal wastewater treatment has been reported to induce significant changes in microbial communities [49]. Similarly, the microalgal treatment in this study showed drastic changes in bacterial communities during the mixotrophic cultivation phase, relative to the heterotrophic phase. This may be due to sudden changes in the environment or microalgal metabolism [50]. The temperature change could be suggested as a major factor for the changes of RG2, but as *Oligella*, *Treponema*, and *Arcobacter* were reported to grow well at 37 °C [51–53], the effect of temperature was not considered for further analysis. Oxidative burst conditions (production of reactive oxygen species) created by microalgal photosynthesis could have reduced the pathogens, or other enriched bacteria might also have affected pathogen decrease by providing stress or nutrient competition conditions [54–56]. However, as the co-cultivation of microalgae and *Oligella* under light irradiation showed no inhibitory effects, ROS and photoinhibition were not the reason for the decrease of pathogens in the microalgal wastewater treatment process. *Oligella ureolytica* is susceptible to a limited number of antibiotics [50]. However, all *Oligella* spp. in this study (ASV1, ASV8, ASV52, etc.) decreased with microalgal wastewater treatment. Rigorous analyses of the interaction mechanisms between microalgae and pathogens are required. Further study on absolute abundances of the microbial communities in the piggery wastewater and their changes will be beneficial for elucidating the cyclic changes observed in Fig. 4B. Interestingly, these four bacteria were found in both the control and treatment groups. In other words, the *Oligella*-inhibitory bacteria were also present in the control group, but they could not suppress *Oligella* growth. Furthermore, a negative correlation between algal growth and *Oligella* (Figure S5) was relatively less significant than the negative correlation between *Oligella* and these bacteria (Figure S6 and Table S4). Therefore, the major reason for the changes in group II and III bacteria was attributed to the effect of *Coelastrella* sp.-associated bacteria and environmental factors, respectively.

## Conclusion

The present study treated undiluted raw piggery wastewater using microalgae at a pilot scale. Most of the nutrients were removed efficiently, and the clarity of wastewater was enhanced. Amplicon sequencing followed by diversity and statistical analyses revealed that a dynamic bacterial community change was induced by inoculating microalgae into the wastewater. RG2 pathogenic taxa decreased only in microalgae-treated PBR. A validation test performed by co-culturing microalgae and *Oligella* sp. (the most abundant pathogenic bacterium) and further network analysis showed that the decrease in RG2 was not a direct effect of *Coelastrella* but was induced by microalgae-associated bacteria. Several of these bacteria were confirmed to inhibit *Oligella* in a validation experiment. Further studies are required, such as continuous annual operation, which could help demonstrate the feasibility of microalgal piggery wastewater treatment combined with the pathogen monitoring of the effluents to reduce public health risks.

## Supplementary Information

**Additional file 1: Figure S1.** Results of next-generation sequencing in family level in both control and microalgae treated group. Data are summarized for 30 most abundant genera and noted as legends. **Figure S2.** Results of next-generation sequencing in order level in both control and microalgae treated group. Data are summarized for 30 most abundant genera and noted as legends. **Figure S3.** Results of next-generation sequencing in class level in both control and microalgae treated group. Data are summarized for 30 most abundant genera and noted as legends. **Figure S4.** Phylogenetic tree of isolated *Oligella* sp. and ASVs assigned as *Oligella* genus constructed by the neighbor-joining method. **Figure S5.** Scatter plot, trend line, 95% confidence band, and 95% prediction band between algal growth and relative abundance of ASV1 (*Oligella*). **Figure S6.** Scatter plot between relative abundances of algae-positive bacteria and ASV1 (*Oligella*). **Table S1.** Compositions and physicochemical properties of the raw wastewater, effluent of control (aerobic digestion), and microalgal treatment, respectively. The effluents were analyzed by using culture broth after 96 hours of treatment. **Table S2.** Summary of non-metric multidimensional scaling (NMDS) results with PERMANOVA test indicating significance level of *: *P* < 0.05 and **: *P* < 0.01. **Table S3.** Inhibitory zone diameters in range and mean with standard deviation (SD) values induced by various microorganisms to *Oligella* sp. The tests were quadruplicated. **Table S4.** Correlation, *p*-value and Benjamini-Hochberg adjusted *p*-value of Pearson correlation between *Oligella* and algal growth, *Brevundimonas*, *Sphingopyxis*, and *Stenotrophomonas*.

## Data Availability

The raw sequences and metadata were submitted to the National Center for Biotechnology Information (NCBI) Sequence Read Archive (SRA). The project number is PRJNA757236.

## Abbreviations

RGs: Risk groups
COD: Chemical oxygen demand
PBR: Photobioreactor
ASV: Amplicon sequence variants
TP: Total phosphorus
DCW: Dry cell weight
PCoA: Principal coordinates analysis
db-RDA: Distance-based redundancy analysis
TSB: Tryptic soy broth
TSA: Tryptic soy agar
ORP: Oxidation-reduction potential
EC: Electrical conductivity
TDS: Total dissolved solids
