# Supplementary material for: Extra benefit of microalgae in raw piggery wastewater treatment: pathogen reduction
Source: Microbiome. 2022 Aug 31;10:142. doi: 10.1186/s40168-022-01339-3 (PMC9429445; doi:10.1186/s40168-022-01339-3)
Supplement: Supplementary file 2 — Additional file 1: Figure S1. Results of next-generation sequencing in family level in both control and microalgae treated group. Data are summarized for 30 most abundant genera and noted as legends. Figure S2. Results of next-generation sequencing in order level in both control and microalgae treated group. Data are summarized for 30 most abundant genera and noted as legends. Figure S3. Results of next-generation sequencing in class level in both control and microalgae treated group. Data are summarized for 30 most abundant genera and noted as legends. Figure S4. Phylogenetic tree of isolated Oligella sp. and ASVs assigned as Oligella genus constructed by the neighbor-joining method. Figure S5. Scatter plot, trend line, 95% confidence band, and 95% prediction band between algal growth and relative abundance of ASV1 (Oligella). Figure S6. Scatter plot between relative abundances of algae-positive bacteria and ASV1 (Oligella). Table S1. Compositions and physicochemical properties of the raw wastewater, effluent of control (aerobic digestion), and microalgal treatment, respectively. The effluents were analyzed by using culture broth after 96 hours of treatment. Table S2. Summary of non-metric multidimensional scaling (NMDS) results with PERMANOVA test indicating significance level of *: P < 0.05 and **: P < 0.01. Table S3. Inhibitory zone diameters in range and mean with standard deviation (SD) values induced by various microorganisms to Oligella sp. The tests were quadruplicated. Table S4. Correlation, p-value and Benjamini-Hochberg adjusted p-value of Pearson correlation between Oligella and algal growth, Brevundimonas, Sphingopyxis, and Stenotrophomonas. [file 40168_2022_1339_MOESM1_ESM.docx]

Supplementary materials

Extra benefit of microalgae in raw piggery wastewater treatment: pathogen reduction

Sang-Ah Lee ^1, 2, 3†^, Minsik Kim ^1†^, Hee-Sik Kim ^1, 2^, and Chi-Yong Ahn ^1, 2^*

^1^ Cell Factory Research Center, Korea Research Institute of Bioscience and Biotechnology (KRIBB), Daejeon 34141, Republic of Korea

^2^ Department of Environmental Biotechnology, KRIBB School of Biotechnology, University of Science and Technology (UST), Daejeon 34113, Republic of Korea

^3^ Environmental Safety Group, Korea Institute of Science and Technology (KIST) Europe, Saarbrücken 66123, Germany

* Corresponding author

Chi-Yong Ahn

Email: [cyahn@kribb.re.kr](mailto:cyahn@kribb.re.kr)

Phone: +82-42-860-4329

^†^ These authors contributed equally to this work.

Table S1. Compositions and physicochemical properties of the raw wastewater, effluent of control (aerobic digestion), and microalgal treatment, respectively. The effluents were analyzed by using culture broth after 96 hours of treatment.

| **Components** | **Raw wastewater** | **Control effluent** | **Treatment effluent** |
| --- | --- | --- | --- |
| NH_3_-N (mg L^-1^) | 770 | 676 | 153 |
| Chemical oxygen demand (COD) (mg L^-1^) | 3100 | 2380 | 680 |
| Total dissolved solids (TDS) (mg L^-1^) | 4840 | 3650 | 4190 |
| Total phosphorus (TP) (mg L^-1^) | 77.2 | 37.8 | 6.95 |
| Boron (mg L^-1^) | 1.01 | 0.86 | 0.39 |
| Sodium (mg L^-1^) | 146 | 297 | 302 |
| Magnesium (mg L^-1^) | 1.65 | 4.22 | 0.695 |
| Potassium (mg L^-1^) | 1070 | 758 | 639 |
| Calcium (mg L^-1^) | 0.95 | 0.685 | 1.87 |
| Iron (mg L^-1^) | 6.28 | 3.42 | 2.15 |
| Cobalt (mg L^-1^) | 0.01 | 0.014 | 0.0125 |
| Copper (mg L^-1^) | 0 | 0.0235 | 0.0475 |
| Molybdenum (mg L^-1^) | 0.9 | 0.12 | 0.105 |
| **Properties** |  |  |  |
| Oxidation-reduction potential (ORP) (mV) | 58 | 40 | 139 |
| Salinity (‰) | 0.2 | 0.35 | 0.43 |
| Conductivity (μS/cm) | 9470 | 6940 | 8390 |
| pH | 8.64 | 9.05 | 7.47 |
| **Biomass** |  |  |  |
| Microalgal dry cell weight (DCW) (g L^-1^) | 0 | 0 | 1.6 |

Table S2. Summary of non-metric multidimensional scaling (NMDS) results with PERMANOVA test indicating significance level of *: *P* < 0.05 and **: *P* < 0.01.

| Factors | NMDS1 | NMDS2 | R^2^ | *P*-value | Significance |
| --- | --- | --- | --- | --- | --- |
| Temperature | 0.8636 | 0.5042 | 0.9254 | 0.0319 | * |
| pH | -0.5523 | -0.8337 | 0.9844 | 0.0556 |  |
| DCW | 0.6272 | 0.7788 | 0.9983 | 0.0167 | * |
| NH_3_ | -0.8637 | -0.5040 | 0.9867 | 0.0208 | * |
| COD | -0.7293 | -0.6842 | 0.9665 | 0.0431 | * |
| ORP | 0.2446 | 0.9696 | 0.9647 | 0.0083 | ** |
| EC | 0.8760 | 0.4823 | 0.8755 | 0.0347 | * |
| Salinity | 0.8431 | 0.5378 | 0.8883 | 0.0597 |  |
| TDS | 0.8427 | 0.5384 | 0.9216 | 0.0458 | * |
| B | -0.8411 | -0.5409 | 0.9463 | 0.0167 | * |
| Na | 0.9598 | 0.2809 | 0.4018 | 0.4222 |  |
| Mg | -0.5887 | -0.8084 | 0.9312 | 0.0861 |  |
| TP | -0.9301 | -0.3672 | 0.8837 | 0.0167 | * |
| K | -0.8350 | 0.5503 | 0.7042 | 0.2028 |  |
| Ca | 0.8003 | 0.5996 | 0.9423 | 0.0153 | * |
| Fe | -0.9484 | 0.3170 | 0.7045 | 0.1625 |  |
| Co | 0 | 0 | 0 | 1.0000 |  |
| Cu | 0.6531 | -0.7572 | 0.4034 | 0.5000 |  |
| Mo | -0.0228 | 0.9997 | 0.9732 | 0.1667 |  |

Table S3. Inhibitory zone diameters in range and mean with standard deviation (SD) values induced by various microorganisms to *Oligella* sp. The tests were quadruplicated.

| Species | Range (mm) | Mean ± SD (mm) |
| --- | --- | --- |
| *Brevundimonas terrae* | 11.50 – 15.05 | 13.44 ± 1.48 |
| *Stenotrophomonas ginsengisoli* | 14.15 – 19.15 | 15.84 ± 2.33 |
| *Stenotrophomonas koreensis* | 10.50 – 17.10 | 14.01 ± 2.73 |
| *Sphingopyxis panaciterrae* | N.D. |  |
| *Coelastrella* sp. | N.D. |  |

Table S4. Correlation, *p*-value and Benjamini-Hochberg adjusted *p*-value of Pearson correlation between *Oligella* and algal growth, *Brevundimonas*, *Sphingopyxis*, and *Stenotrophomonas*.

| X axis | Y axis | Correlation | *p*-value | *p_BH* |
| --- | --- | --- | --- | --- |
| ASV1 *Oligella* | Algal growth | -0.84591 | 9.74E-06 | 1.24E-04 |
| ASV1 *Oligella* | ASV93 | -0.84776 | 8.89E-06 | 1.15E-04 |
| ASV1 *Oligella* | ASV120 | -0.80058 | 6.58E-05 | 5.57E-04 |
| ASV1 *Oligella* | ASV115 | -0.87537 | 1.97E-06 | 3.32E-05 |
| ASV1 *Oligella* | ASV145 | -0.86115 | 4.45E-06 | 6.57E-05 |
| ASV1 *Oligella* | ASV220 | -0.95043 | 1.57E-09 | 7.04E-08 |


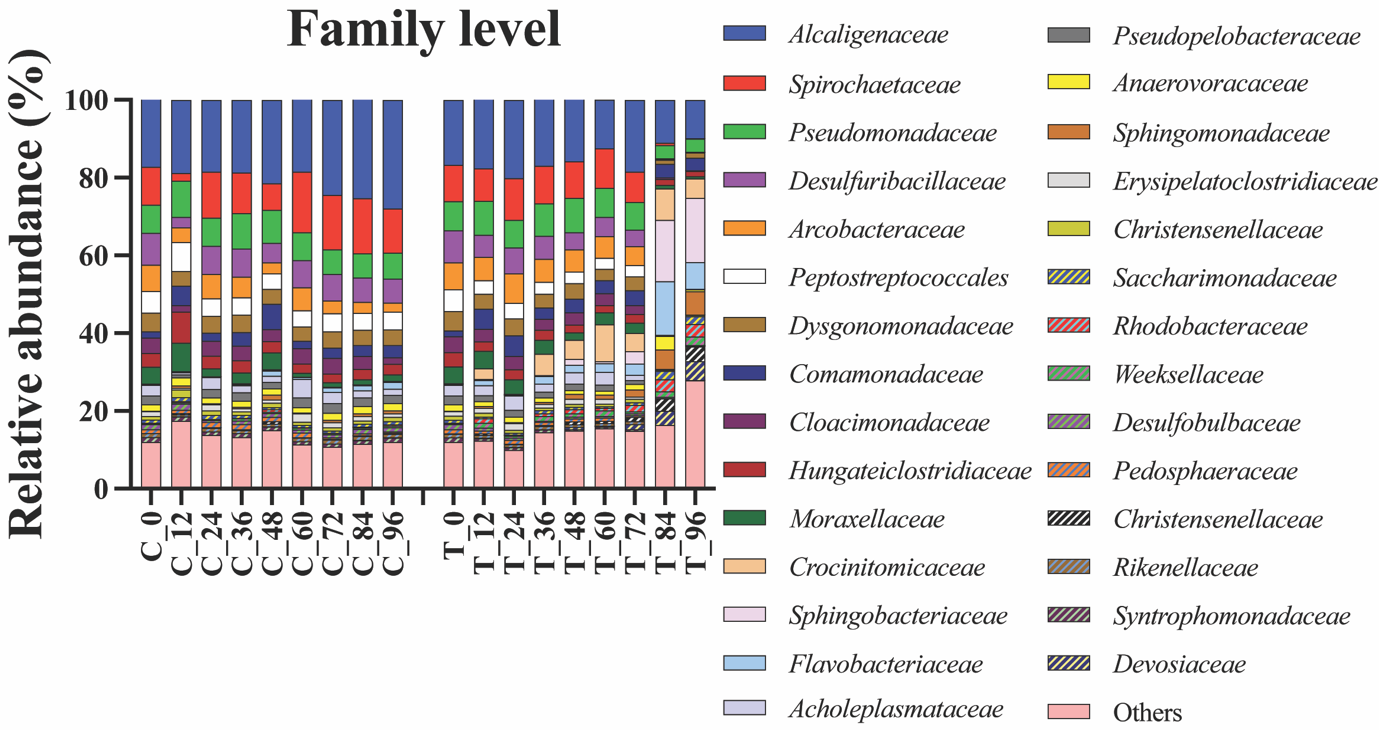


Fig. S1. Results of next-generation sequencing in family level in both control and microalgae treated group. Data are summarized for 30 most abundant genera and noted as legends.


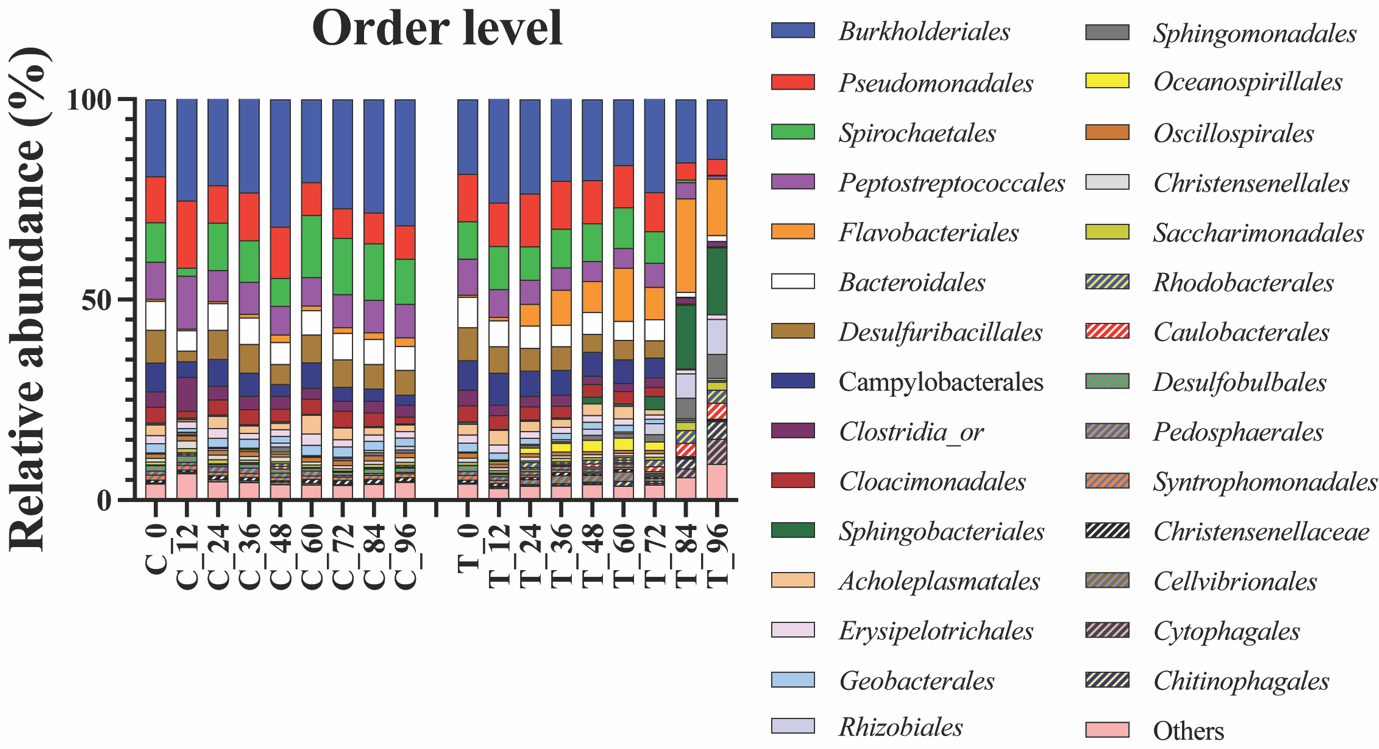


Fig. S2. Results of next-generation sequencing in order level in both control and microalgae treated group. Data are summarized for 30 most abundant genera and noted as legends.


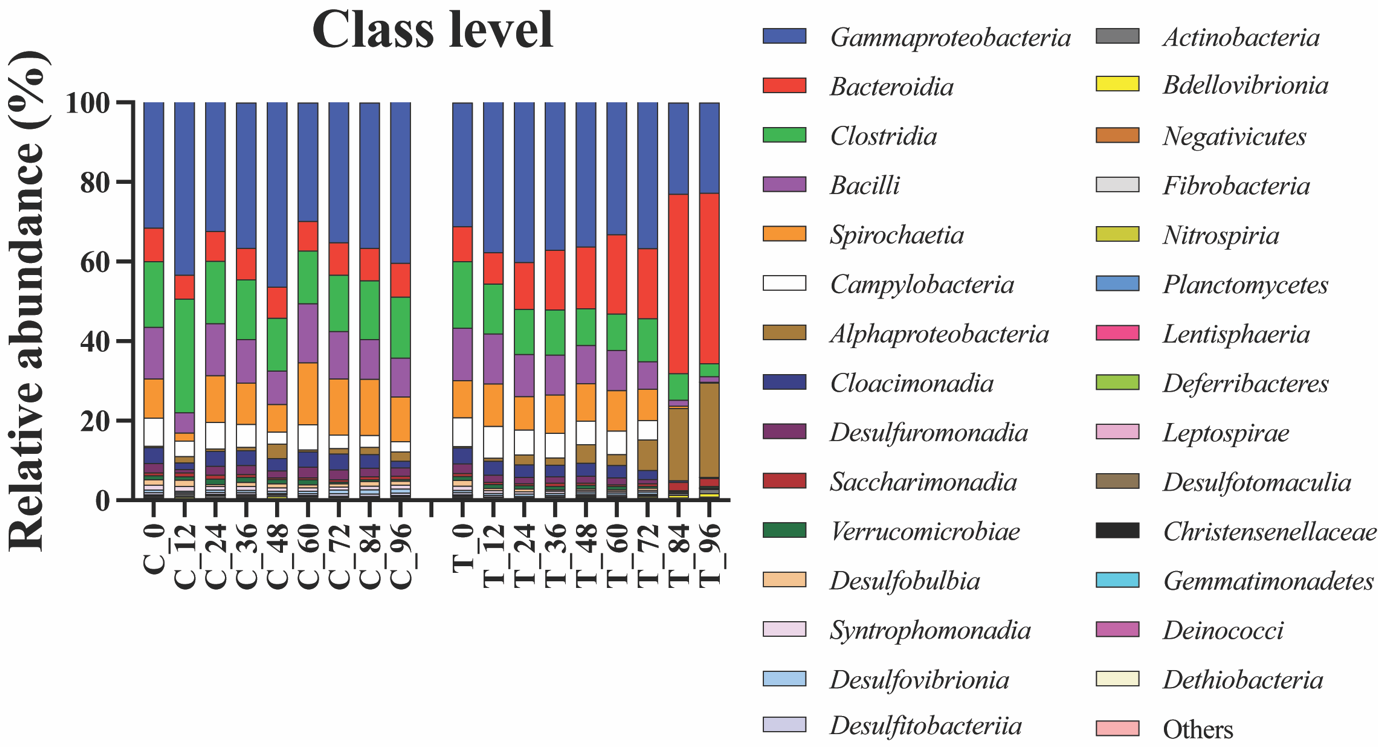


Fig. S3. Results of next-generation sequencing in class level in both control and microalgae treated group. Data are summarized for 30 most abundant genera and noted as legends.


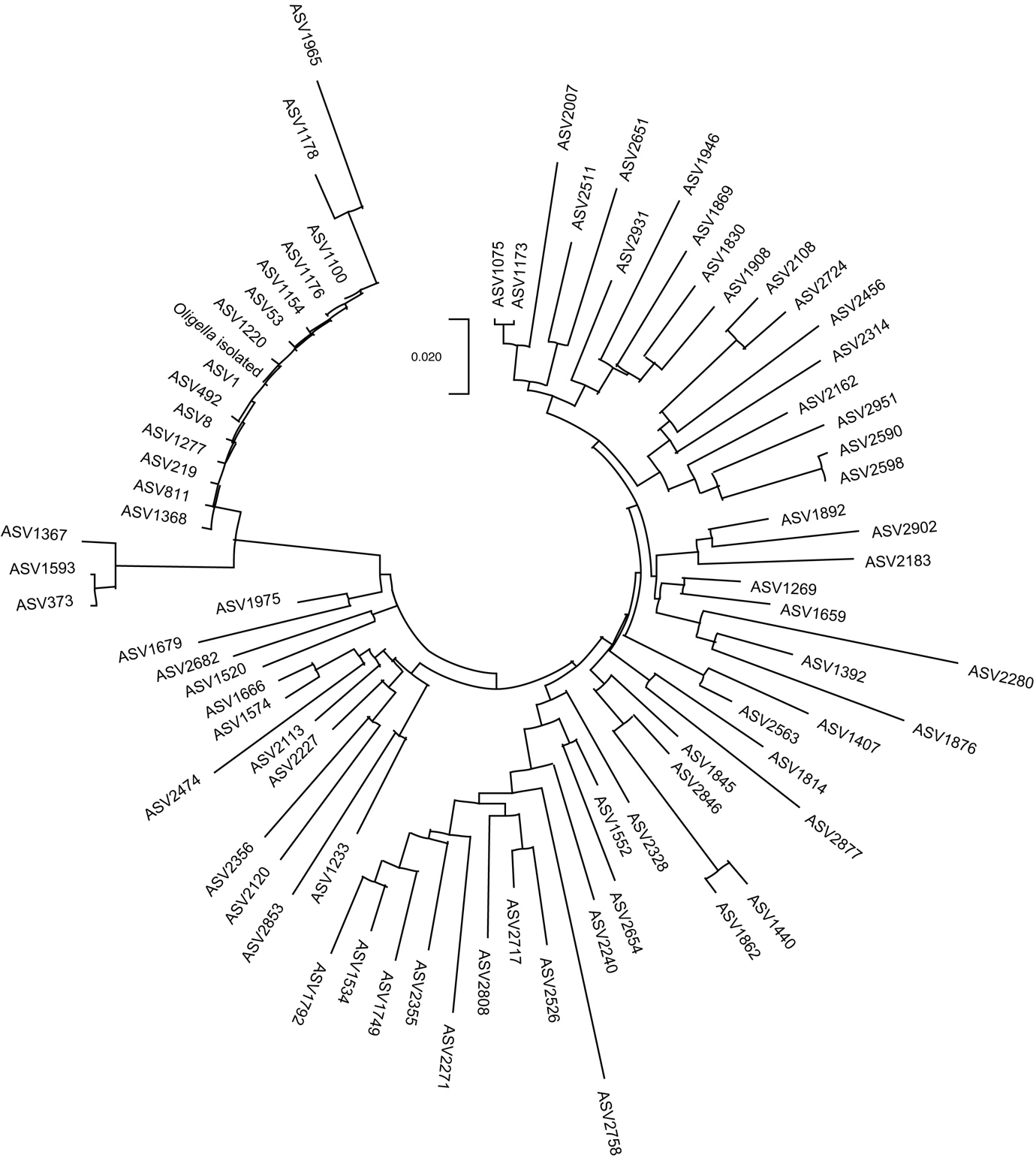


Fig. S4. Phylogenetic tree of isolated *Oligella* sp. and ASVs assigned as *Oligella* genus constructed by the neighbor-joining method.


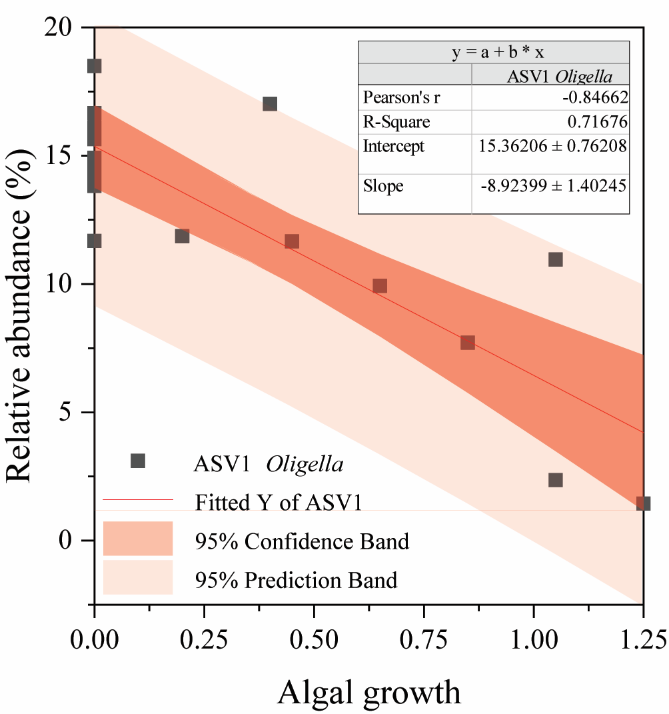


Fig. S5. Scatter plot, trend line, 95% confidence band, and 95% prediction band between algal growth and relative abundance of ASV1 (*Oligella*).


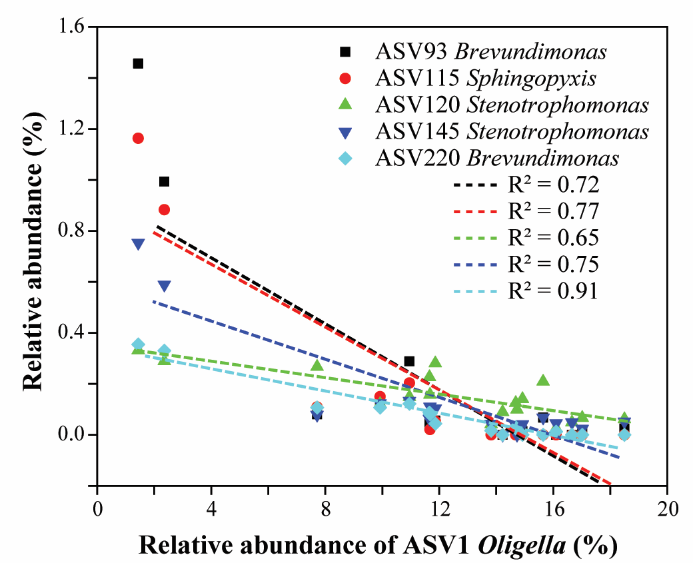


Fig. S6. Scatter plot between relative abundances of algae-positive bacteria and ASV1 (*Oligella*).
